# Attractive running environments for all? A cross-sectional study on physical environmental characteristics and runners’ motives and attitudes, in relation to the experience of the running environment

**DOI:** 10.1186/s12889-019-6676-6

**Published:** 2019-04-02

**Authors:** Ineke Deelen, Mark Janssen, Steven Vos, Carlijn B. M. Kamphuis, Dick Ettema

**Affiliations:** 1 Department of Human Geography and Spatial Planning, Faculty of Geosciences, Utrecht University, Utrecht, The Netherlands; 2 School of Sport Studies, Fontys University of Applied Sciences, Eindhoven, The Netherlands; 3 Department of Industrial Design, Eindhoven University of Technology, Eindhoven, The Netherlands; 4 Department of Movement Sciences, KU Leuven, Leuven, Belgium; 5 Department of Interdisciplinary Social Science, Faculty of Social and Behavioural Sciences, Utrecht University, Utrecht, The Netherlands

**Keywords:** Running, Experience, Physical environment, Motives and attitudes, Attractive design, Restorative capacity

## Abstract

**Background:**

Running has become one of the most popular sports and has proven benefits for public health. Policy makers are increasingly aware that attractively designed public spaces may promote running. However, little is known about what makes a running environment attractive and restorative for runners and to what extent this depends on characteristics of the runner. This study aims to investigate 1) to what extent intrapersonal characteristics (i.e. motives and attitudes) and perceived environmental characteristics (e.g. quality of the running surface, greenness of the route, feelings of safety and hinderance by other road users) are associated with the perceived attractiveness and restorative capacity of the running environment and 2) to what extent the number of years of running experience modify these associations.

**Methods:**

Cross-sectional data were collected through the online Eindhoven Running Survey 2015 (ERS15) among half marathon runners (*N* = 2477; response rate 26.6%). Linear regression analyses were performed for two outcomes separately (i.e. perceived attractiveness and perceived restorative capacity of the running environment) to investigate their relations with motives and attitudes, perceived environmental characteristics and interactions between perceived environmental characteristics and number of years of running experience.

**Results:**

Perceived environmental characteristics, including green and lively routes and a comfortable running surface were more important for runners’ evaluation of the attractiveness and restorative capacity of the running environment than runners’ motives and attitudes. In contrast to experienced runners, perceived hinder from unleashed dogs and pedestrians positively impacted the attractiveness and restorative capacity for less experienced runners.

**Conclusions:**

Perceived environmental characteristics were important determinants of the attractiveness and restorative capacity of the running environment for both novice and experienced runners. However, green and lively elements in the running environment and hinderances by cars were more important for less experienced runners. In order to keep novice runners involved in running it is recommended to design comfortable running tracks and routes and provide good access to attractive, green and lively spaces.

## Background

Increasing participation in sport and physical activity is an important health policy objective [1–3]. Sports participation is associated with positive benefits for physical and mental health and well-being [4, 5]. In particular, positive effects have been found for running as an integral part of an active and healthy lifestyle [6–9]. In recent decades, running has rapidly become more popular and accessible to many people. In the Netherlands, running is one of the most practiced sports [10]. Among Dutch adults between the ages of 20–79 years, 13.2% of men and 11.3% of women reported running at least once a month in 2012 [10, 11]. These figures are similar to data from other Western countries [12]. Running has increasingly become a ‘lifestyle sport’, with runners focusing on improving their health, wellbeing and image [13]. Currently, more and more runners participate individually, in informal groups, in running events or in low-threshold exercise (‘start to run’) programmes instead of in traditional sports clubs focusing on competition [14, 15]. The growing popularity of recreational running can be understood in light of the individualization of sports participation. This has resulted in an increased popularity of types of sports activities that are informal and flexible in time and space and which have increased more rapidly than sports participation in traditional organized sports clubs [1, 16–19]. Informal and flexible sports activities have been referred to as ‘light’ sports activities that take place in light sports settings, in contrast to the ‘heavy’ or organized settings of sports clubs [19, 20–22]. As a result, recreational running have become increasingly diverse and different running subcultures and identities have emerged [23].

The increased popularity of running individually or in informal groups has also led to a greater variety of geographical locations used, including public spaces such as parks and natural environments [16, 19, 24–28]. Various studies showed that some environments may facilitate and strengthen the health benefits of running, whereas other environments hinder running. Thus, it matters where (e.g. at what geographical location, indoors or outdoors or at which running surface) an individual runs [29–31]. Policy makers increasingly recognize that the built environment can function as an important condition for active living environments. Municipalities aspire to design cities that encourage people to be more physically active [32–34], for example by developing attractive urban running trails and routes [19, 35].

However, little is known about what specific environmental characteristics make a running environment experienced as attractive and restorative by runners and to what extent this experience depends on the personal characteristics of the runner. What makes a public space an attractive environment for specific types of runners, one that invites people to run and keep running? Understanding this is important for several reasons. Attractive environments may promote participation in sport and physical activity, including running [32, 36, 37], which contributes to a more physically active and healthy population [6–9]. These positive health effects of attractive environments for sports participants have been well documented. For instance, exercising in nature or green environments, also referred to as ‘green exercise’ [38], has been associated with greater physical and mental health benefits, including lower blood pressure, stress reduction, and with improving mood, self-esteem, perceived health and wellbeing [39–44]. In addition, the restorative capacity of the environment increases wellbeing and contributes to the adherence of healthy behaviours such as running. Furthermore, attractively designed public spaces contribute to pleasurable and liveable urban environments and can have benefits beyond health, such as the environmental sustainability and economic vitality of cities [33, 45]. Therefore, designing attractive and restorative environments increases the positive experiences of users. Finally, providing more insight into the experiences of different groups of less and more experienced runners may help policy makers to make informed choices with regard to designing public spaces and professionals to gain attention for the promotion of healthy urban living.

To understand the factors that determine how the running environment is experienced, this study applies a socio-ecological framework, which is frequently used in studies on physical activity [46] and sports participation [47, 48]. According to the socio-ecological approach, there are multiple influences on specific health behaviours, including factors on the intrapersonal, interpersonal and environmental level. All influences on health behaviours potentially interact across these different levels [46]. In this paper, we particularly focus on the interplay between intrapersonal and environmental characteristics and how these relate to the experience of the running environment. Although there is much theoretical evidence for the importance of interactions on the intrapersonal and environmental level, empirical evidence is rather limited and results differ greatly depending on the specific interactions studied [49]. Therefore, more insight into the role of these interactions in the context of one specific type of sport (i.e. running) adds to the current body of literature.

Intrapersonal factors, such as motivation, the reasons why a person participates in sport, have an important impact on persistence in sports participation and the frequency of participation [50, 51]. Research on running has shown that the majority of the European running population runs because of health goals, such as getting fit (54%) or losing weight (40%). Other motives are having fun (22%) and/or relieving stress (21%) [12]. However, as mentioned earlier, runners are a very heterogeneous group [14, 15, 27, 52, 53]. For example, runners differ regarding their motivations and goals related to health, competitiveness and sociality [52, 53], and their meanings may be experienced both negatively and positively [27]. Furthermore, the level of competitiveness and experience in running can explain differences between types of runners. Running increasingly loses its competitive image and most runners now belong to a group of recreational ‘casual’ runners who are unattached to a running club. For many of them, “‘completing’ is much more preferred than ‘competing’” [54]. In contrast to competitive runners in traditional ‘heavy’ settings such as sports clubs, this group of recreational ‘light’ runners is still underexposed in research [27]. Based on a qualitative study which included accompanied runs and interviews with 20 recreational runners in London, Hitchings and Latham [23] found that while the majority of the running activities of these runners are performed alone, they do find ‘sociality’ and the presence of other runners important. Especially when this sociality is characterized as ‘loose’ and engagement in running activities can take place without obligations or a strong sense of belonging. Interestingly, not all runners in this study perceived their running as a ‘sport’ and for them “doing running’ does not require becoming a ‘runner’” [23]. However, Shipway et al. [6] showed in a study among 25 long distance runners who trained at least five times a week for distances ranging from 5 km to marathon, that more dedicated and ‘serious’ competitive runners may have different motives and preferences, such as a strong desire for a healthy lifestyle. The authors found that runners’ desires for a healthy lifestyle and wellbeing included, − besides a strong focus on the ‘running body’ -, both positive aspects related to the importance of seeking self-esteem and confirmation through running, as well as negative aspects such as exercise addiction and the need to exercise [6]. In addition, differences in running motives and attitudes may also be related to runners’ years of experience in running. For example, in a quantitative Danish study (*N* = 4052), it was found that runners with three or fewer years of running experience focused more on health reasons, whereas runners who were running for eight or more years were more frequently running for ‘the love of it’ or for social reasons [55].

In addition to intrapersonal factors, the influence of the physical environment on health and healthy lifestyles including physical activity has been studied extensively in the public health and physical activity domains [34, 56–58]. Objectively measured environmental factors, such as street design, land use mix, street connectivity, access to and availability of facilities, − such as shops and recreational or sports facilities, proximity of green spaces -, population density and socioeconomic status of the neighbourhood are associated with different types and intensities of physical activity [37, 59–62] and sports participation [28, 47, 63]. In addition, perceptions of the physical environment, including perceived safety and attractiveness, are related to sports participation [63, 64]. However, less is known about the environmental correlates of running (that are, the characteristics of the physical environment that may impact on running behaviour). Although running significantly differs from walking regarding pace, intensity, bodily experience and spatial reach, studies found indications that recreational walking and running may have similar environmental correlates, because recreational walkers and runners use the same public spaces [36]. Perceived characteristics of the physical environment associated with recreational walking include perceived safety, aesthetics, quality of the walking infrastructure and attractiveness of the environment (e.g. presence of cafes and other people and quiet and green areas) [58, 65–67]. An indication of the importance of the physical environment for encouraging running was provided by Titze et al. [68]. This study showed that women who perceived themselves as less healthy and who lived in an unattractive neighbourhood were more likely to quit running. Factors including an attractive neighbourhood and social support were likely to play a key role in encouraging running [68].

While many studies found evidence for the importance of objective characteristics of the physical environment for physical activity and sports participation, fewer focused on how physical environmental characteristics affects how the running environment is experienced and how this differs for different types of runners [27, 69]. Since ‘the mobility turn’ in the social sciences, more attention has been paid to so called embodied experiences. For example, Cresswell [70] introduced a more holistic view of mobility, wherein the complex interplay between movement, experience and representation (or meaning) is central, instead of the perception of mobility as a ‘getting from A to B’. Running can therefore be seen as an interaction between the body, senses and the environment. The experiences of the body are lived through the senses. Touching, smelling, feeling, hearing and seeing allows runners to run safely, choose and recognize terrain, adapt pace and take other runners and road users into account [27, 71, 72]. These experiences of runners can be positive and negative, pleasurable and painful [73] and are therefore likely to influence running behaviour (e.g. distance, pace and frequency), choices for specific surfaces or running environments, as well as the perseverance of running.

Several studies, all targeting different groups of runners showed that various running surfaces or terrains are experienced differently by different runners (e.g. samples with experienced and less experienced [19], novice runners participating in a start to run programme [36], recreational runners/joggers [27], and middle distance runners [72, 74]) and impact whether the running environment is evaluated as attractive. For example, Borgers et al. [19] held structured face-to-face interviews with 546 randomly selected runners at various bark running tracks, (i.e. informal running facilities in the public space consisting of paths with soft surfaces), and found that these running tracks were highly valued among runners because of injury prevention. These running facilities were experienced as attractive by unorganized recreational runners, and showed potential to reach runners at different levels and stimulate people to start running [19]. In addition, Bodin and Hartig [29] found that experienced runners prefer green environments over urban settings as they offer more fascination and help escape from daily hassles.

The above literature has shown a great variation in the motives and experiences of different types of runners. It is likely that these different types of runners also differ in their requirements and experiences regarding the running environment and therefore perceive the attractiveness or restorative capacity of the environment differently. Whereas most studies on running focus on one specific type of runners (e.g. competitive long distance runners or unorganized recreational or ‘casual’ runners or joggers) and are based on rich qualitative data with small sample sizes [23, 27, 72, 74], studies investigating differences between various types of runners based on larger and representative data sets are lacking. In this paper, we specifically distinguish between significant groups of experienced and less experienced runners in order to learn more about the preferences and experiences of novice runners. We expect that novice or inexperienced runners may differ from experienced runners with regard to their running motives and attitudes and their preferences in terms of running distance, interactions with other road users or the running surface [19, 36]. We expect, for example, that the presence of other road users, such as cars, cyclists and unleashed dogs, may affect whether novice runners experience their running environment as attractive, whereas experienced runners know how to address this and are less affected. From a public health and sports promotion point of view, greater insight into the experiences of different groups of runners is important to develop targeted and effective policy interventions, particularly at the level of urban planning and design. This contributes to a better understanding of how novice runners may be better encouraged and facilitated to keep active and involved in sport [69]. This study aims to 1) investigate to what extent characteristics on the intrapersonal level (i.e. motives and attitudes towards running) and the physical environmental level (i.e. perceived constraints by other road users, feelings of safety and quality and characteristics of the running surface and routes) are associated with the perceived attractiveness and perceived restorative capacity of the running environment and 2) to what extent the number of years of running experience modify the association between perceived environmental characteristics and attractiveness and restorative capacity of the running environment.

## Methods

### Study design and respondents

For this cross-sectional study, the Eindhoven Running Survey 2015 (ERS15) was used to collect data among participants of the Eindhoven Marathon running event in October 2015. The survey questions were based on the Eindhoven Running Survey 2014 (ERS14) and used in previous studies [52, 53, 75]. For the current study, a sub-dataset containing only those runners who participated in the Half Marathon Eindhoven 2015 (21.1 k) was used. Consistent with Janssen et al. [52], half marathon runners were selected because of the heterogeneous characteristics of this group of participants, which included both highly experienced and less experienced runners. At registration for the event, all participants agreed that they could be approached for an online questionnaire after the event. After finishing the half marathon, all registered participants (*N* = 9314) received an email with an introductory letter and a web link to the online questionnaire. The introduction letter informed them about the purpose of the study and the guarantee that the data would be processed anonymously and in accordance with the ethical principles of the Declaration of Helsinki. After clicking on the link to the questionnaire, respondents were given the choice to end or to continue with the questionnaire. They also were given the opportunity to declare that they do not want to be approached more often. The questionnaire started with a similar announcement about the purpose of the study and privacy. After the announcement, the respondents again had to confirm that they wanted to start the questionnaire. None of the questions were required to fill in. In total, 2477 participants fully completed the questionnaire (response rate of 26.6%). The socio-demographic background of the respondents was comparable to other samples in previous large-scale running studies in Western Europe [12].

### Measures

Consistent with the socio-ecological approach, the online questionnaire consisted of blocks with questions representing socio-demographic and running-related characteristics, motives and attitudes towards running, and characteristics of the running environment.

#### Outcome variables: perceived attractiveness and restorative capacity of the running environment

Two dependent variables were analysed: perceived attractiveness of the running environment and perceived restorative capacity of the running environment. Both variables were measured with a single item and scored on a five-point Likert scale ranging from 1 (totally disagree) to 5 (totally agree). Respondents were asked to rate the following two statements: ‘the environment through which my running route passes is attractive’ and ‘the environment through which my running route passes is relaxing’. This approach of measuring attractiveness and restorative capacity of the environment in single-item measures is consistent with previous research on this and related topics, including satisfaction, wellbeing, preferences for places and experience of place qualities [36, 76, 77].

#### Intrapersonal characteristics: motives and attitudes, and number of years of running experience

The first set of independent variables included intrapersonal characteristics, namely motives and attitudes towards running. In total, 25 items on motives and attitudes towards running were measured (based on Janssen et al. (2017) [52]). On a five-point Likert scale ranging from 1 (totally disagree) to 5 (totally agree), runners were asked to rate the extent to which they agreed with statements. All items were included in a principal component analysis (PCA) with orthogonal varimax rotation (EVA = 59.1%). As a result, the following five psychographic components were formed: 1) bodily and mental advantages of running (e.g. running gives me energy or running is good for my health), 2) identification with running (e.g. I am proud to be a runner or I feel myself a real runner), 3) practical advantages of running (e.g. I can practise running anytime, anywhere), 4) individual motives for quitting (e.g. I would quit running if I get injured or if my spare time would decrease) and 5) social motives for quitting (e.g. I would quit running if my trainer quits or if my running friends quit). Table 1 shows the components including the number of items, Cronbach’s alphas, average scores and standard deviations. We included number of years of running experience as a moderator in the analyses and we distinguished between running < 1 year (novice runners), 1–5 years (moderate experienced runners) and >  5 years (experienced runners).

**Table 1:** Internal consistency on motives and attitudes toward running (*N* = 2477)

Motives and attitudes toward running	Items	Cronbach’s alpha (based on standardized items)
Bodily and perceived advantages of running	4	0.862
Identification with running	8	0.796
Practical advantages of running	5	0.753
Individual motives for quitting	5	0.688
Social motives for quitting	3	0.912

#### Perceived environmental characteristics

The second set of independent variables included perceived environmental characteristics (based on Ettema [36]). Respondents were asked to indicate on a five-point Likert scale ranging from 1 (totally disagree) to 5 (totally agree), to what extent they agreed with 10 statements on constraining/negative and encouraging/positive environmental features. Constraining items included interactions with pedestrians, cyclists, cars and unleashed dogs (e.g. I am hindered by unleashed dogs on my running route) and experiences with (verbal) harassment or threats and poor street lighting. Encouraging items included a comfortable running surface, a mostly green running route and a lively route (i.e. a vibrant or vivid running environment where other people are present and activities are going on).

#### Potential confounders

We controlled our analyses for socio-demographics and running-related characteristics. Socio-demographics included age, sex and education. Education was classified into three levels based on the self-reported highest level of completed education (lower, middle or higher education). Running-related characteristics included distance monitoring of the running route (yes/no); use of monitoring devices (watch yes/no, app yes/no) and organizational running context (individual, friends/small group or athletics club). Monitoring variables were included as confounders because the use of apps and watches have been frequently used by less experienced runners and have been associated with being more physically active and feeling and behaving healthier and may therefore influence the motives and attitudes of runners [78, 79]. In addition, monitoring devices, particularly those with a GPS feature, are one of the most frequently used functions of monitoring devices by runners [52, 53] and may act as a proxy for awareness of the running environment, as runners may choose specific running routes based on their devices.

### Statistical analyses

All analyses were conducted in SPSS 24.0. Descriptive statistics on respondents’ socio-demographic, running related, motivational and perceived environmental characteristics were examined. Chi-squares and analyses of variance (ANOVA) were conducted to test for significant differences regarding these characteristics between respondents with different years of running experience (i.e. < 1, 1–5 or >  5 years). Subsequently, two linear regression analyses (Enter method) were performed for perceived attractiveness (outcome variable 1) and perceived restorative capacity of the running environment (outcome variable 2) to investigate their relationships with potential confounders, motives and attitudes, and perceived environmental characteristics (model 1). To test whether the association of perceived environmental characteristics with the outcomes differed between novice and experienced runners, interactions between perceived environmental characteristics and number of years of running experience were included (model 2).

## Results

### Descriptive results and differences between runners with different years of running experience

Of all respondents, 44.9% had 1 to 5 years of running experience, 42% was experienced (> 5 years) and 13.1% was relatively inexperienced (novice) and started running less than one year ago (Table 2). Novice runners were younger (58% was younger than 35 years old) and more frequently engaged individually in running (71.9%). They scored significantly lower on bodily and mental advantages of running (M = 4.3; SD = 0.5) compared to experienced runners (M = 4.4; SD = 0.5) and on identification with running (M = 3.5; SD = 0.5) than the average of the sample (M = 3.8; SD = 0.5). Novice runners more frequently had individual quitting motives (M = 3.2; SD = 0.7) than the average (M = 2.9; SD = 0.7) and particularly compared to more experienced runners (M = 2.7; SD = 0.7). The average score on attractiveness (M = 4.0; SD = 0.9) and restorative capacity (M = 4.0; 0.8) suggests that runners were quite satisfied with their running environment, although these scores differed significantly for runners with less and more years of experience.

**Table 2:** Descriptive statistics of respondents with different years of running experience

	Total (*N* = 2477)	Novice runners (< 1 y) (*N* = 324; 13.1%)	Moderate experienced runners (1–5 y) (*N* = 1112; 44.9%)	Experienced runners (> 5 y) (*N* = 1041; 42,0%)	*P*-values
Age, *mean (SD)*					
≤ 35 year	32.3	58.0	39.3	16.8	< 0.001
36–45 year	32.5	28.4	37.6	28.3	
≥ 46 year	35.2	13.6	23.1	54.9	
Female (%)	32.5	28.7	39.0	26.8	< 0.001
Education (%)					
Lower or middle	29.6	28.4	30.0	29.4	0.841
Higher	70.4	71.6	70.0	70.6	
Monitoring of distance (%)					< 0.001
Yes	57.3	70.4	62.7	47.6	
No	42.7	29.6	36.3	52.4	
Monitoring via sports watch (%)					< 0.001
Yes	53.0	28.7	51.7	62.0	
No	47.0	71.3	48.3	38.0	
Monitoring via app (%)					< 0.001
Yes	34.7	59.9	39.8	21.3	
No	65.3	40.1	60.2	78.7	
Organizational running setting (%)					< 0.001
Individual	56.6	71.9	58.1	50.2	
Friends, colleagues, small group	32.1	23.1	22.3	24.1	
Athletics club	20.3	5.0	19.6	25.7	
Motives and attitudes, *mean (SD)*					
Bodily and mental advantages of running	4.4 (0.5)	4.3 (0.5)	4.4 (0.5)	4.4 (0.5)	< 0.001
Identification with running	3.8 (0.5)	3.5 (0.5)	3.8 (0.5)	3.8 (0.5)	< 0.001
Practical advantages of running	4.1 (0.5)	4.1 (0.5)	4.1 (0.5)	4.1 (0.5)	0.708
Individual motives for quitting	2.9 (0.7)	3.2 (0.7)	2.9 (0.7)	2.7 (0.7)	< 0.001
Social motives for quitting	1.6 (0.7)	1.6 (0.8)	1.7 (0.7)	1.6 (0.7)	0.064
Experiences of the running environment, *mean (SD)*					
Hinderance by pedestrians	1.7 (0.7)	1.7 (0.7)	1.7 (0.7)	1.7 (0.7)	0.169
Hinderance by cyclists/mopeds	2.0 (1.0)	2.0 (1.0)	2.2 (1.0)	2.0 (1.0)	0.403
Hinderance by cars	2.1 (1.0)	2.0 (1.0)	2.1 (1.0)	2.0 (1.0)	0.044
Hinderance by unleashed dogs	2.2 (1.1)	1.9 (1.0)	2.2 (1.0)	2.3 (1.1)	< 0.001
Hinderance through remarks	1.5 (0.7)	1.5 (0.8)	1.6 (0.7)	1.5 (0.7)	0.41
Hinderance through threats	1.5 (0.7)	1.5 (0.7)	1.5 (0.7)	1.5 (0.6)	0.171
Hinderance through poor lighting	2.6 (1.2)	2.7 (1.2)	2.7 (1.2)	2.5 (1.2)	< 0.001
Comfortable surface	3.6 (0.9)	3.7 (0.9)	3.6 (1.0)	3.6 (0.9)	0.099
Lively route	4.0 (0.8)	4.0 (0.8)	3.9 (0.8)	4.0 (0.8)	0.175
Green route	3.6 (0.9)	3.6 (0.9)	3.5 (0.9)	3.6 (0.9)	0.238
Score on attractiveness and restorative capacity (outcome variables), *mean (SD)*					
Attractiveness	4.0 (0.9)	4.0 (0.9)	3.9 (0.9)	4.1 (0.9)	< 0.001
Restorative capacity	3.9 (0.8)	3.9 (0.8)	3.9 (0.9)	4.0 (0.8)	< 0.001

### Associations with attractiveness of the running environment

Table 3 shows the results of the regression analyses on perceived attractiveness of the running environment (adjusted R^2^ = 0.509 in models 1 and 2). Model 1 shows that runners who valued running highly because of the perceived bodily and mental advantages (β = 0.037; *p* < 0.05) or practical advantages (β = 0.043; *p* < 0.05), perceived their running environment as more attractive. Those who perceived hinderance by pedestrians (β = − 0.049; *p* < 0.01) or cars (β = − 0.038; *p* < 0.05) perceived the running environment as less attractive. Poor lighting (β = 0.037; *p* < 0.05), a comfortable running surface (β = 0.17; *p* < 0.001) and running in a lively (β = 0.33; *p* < 0.001) or mostly green route (β = 0.434; *p* < 0.001) were associated with a more attractive running environment. In model 2, interactions with years of running experienced were added with the most experienced runners (> 5 years) as reference. Hinderance by unleashed dogs was positively associated with perceived attractiveness for novice runners (β = 0.269; *p* < 0.05). A lively route was positively associated with perceived attractiveness among novice runners (β = 0.236; *p* < 0.05) but not among more experienced runners. Finally, a comfortable running surface was important for the perceived attractiveness of the running environment among moderately experienced runners (β = 0.209; *p* < 0.01) but no significant differences were found for novice or experienced runners.

**Table 3:** Linear regression on perceived attractiveness of the running environment (*N* = 2477)

	Model 1 (confounders, motives and attitudes, perceived environmental characteristics)		Model 2 (model 1 + perceived environmental – years of running experience interactions)	
	St. Beta (p)	SE	St. Beta (p)	SE
Constant^a^	0.574*	0.253	1.314	0.744
*Confounders*				
Age (ref = ≥ 46 year)				
≤ 35 year	− 0.016	0.032	− 0.016	0.032
36–45 year	− 0.04*	0.029	− 0.042*	0.029
Male (female = ref)	− 0.016	0.025	− 0.015	0.025
Education (higher = ref)				
Lower or middle	−0.011	0.026	−0.011	0.026
Years of running experience (> 5 year = ref)				
< 1 year	0.009	0.039	− 0.112	0.294
1–5 year	− 0.025	0.026	− 0.1	0.178
Distance monitoring y/n (ref = no)	0.001	0.028	−0.001	0.028
Watch use (ref = no)	0.012	0.039	0.014	0.039
App use (ref = no)	0.014	0.045	0.013	0.045
Organizational context (athletics club = ref)				
Individual	0.050*	0.034	0.052*	0.034
Friends, colleagues, small group	0.011	0.036	0.013	0.036
*Intrapersonal characteristics: motivations and attitudes*				
Bodily and mental advantages of running	0.037*	0.031	0.039*	0.031
Identification with running	−0.015	0.027	−0.019	0.027
Practical advantages of running	0.043*	0.027	0.043*	0.027
Individual motives for quitting	−0.015	0.018	−0.016	0.018
Social motives for quitting	−0.003	0.019	−0.002	0.019
*Perceived environmental characteristics*				
Hinderance by pedestrians	−0.049**	0.021	−0.089	0.158
Hinderance by cyclists/mopeds	−0.006	0.015	0.03	0.119
Hinderance by cars	−0.038*	0.014	0.046	0.112
Hinderance by unleashed dogs	0.011	0.012	−0.287*	0.097
Hinderance through remarks	−0.001	0.022	0.153	0.172
Hinderance through threats	−0.031	0.025	−0.068	0.191
Hinderance through poor lighting	0.037*	0.01	0.126	0.078
Comfortable surface	0.17***	0.013	−0.103	0.101
Lively route	0.33***	0.013	0.161	0.104
Green route	0.434***	0.014	0.606***	0.113
Interactions perceived environment characteristics * years of running experience (ref = > 5 y running experience)				
Pedestrians * < 1 y running experience			0.003	0.064
Pedestrians * 1–5 y running experience			0.048	0.044
Cyclists/mopeds * < 1 y running experience			−0.029	0.048
Cyclists/mopeds * 1–5 y running experience			−0.007	0.033
Cars * < 1 y running experience			−0.041	0.045
Cars * 1–5 y running experience			−0.058	0.031
Unleashed dogs * < 1 y running experience			0.269*	0.041
Unleashed dogs * 1–5 y running experience			0.061	0.025
Remarks * < 1 y running experience			−0.123	0.067
Remarks * 1–5 y running experience			−0.047	0.050
Threats * < 1 y running experience			−0.009	0.077
Threats * 1–5 y running experience			0.056	0.053
Poor lighting * < 1 y running experience			−0.053	0.032
Poor lighting * 1–5 y running experience			−0.05	0.021
Comfortable surface * < 1 y running experience			0.177	0.042
Comfortable surface * 1–5 y running experience			0.209**	0.027
Lively route * < 1 y running experience			0.236*	0.042
Lively route * 1–5 y running experience			−0.034	0.029
Green route * < 1 y running experience			−0.173	0.046
Green route * 1–5 y running experience			−0.078	0.031
*Model fit*				
Adjusted R^2^	0.509		0.509	
SE	0.5607		0.5605	

^a^Constant: Unstandardized Beta instead of Standardized Beta

*Significance < 0.05; **Significance < 0.01; ***Significance < 0.001

### Associations with restorative capacity of the running environment

Table 4 shows the results of the regression analyses on the restorative capacity of the running environment (adjusted R^2^ = 0.599 in model 1 and R^2^ = 0.602 in model 2). Model 1 showed that runners who valued running highly because of perceived bodily and mental advantages (β = 0.041; *p* < 0.05) found their running environment more restorative. Green (β = 0.686; *p* < 0.001) and lively (β = 0.128; *p* < 0.001) running routes and a comfortable surface (β = 0.037; *p* < 0.01) were positively associated with restorative capacity. Hinderance by cars was negatively associated with restorative capacity (β = − 0.040; *p* < 0.01). However, interactions in model 2 revealed that this was more so for novice runners (β = − 0.205; *p* < 0.05) and moderately experienced runners (β = − 0.192; *p* < 0.01) than experienced runners. Furthermore, hinderance by pedestrians was positively associated with a restorative running environment among moderately experienced runners (β = 0.24; *p* < 0.01).

**Table 4:** Linear regression on perceived restorative capacity of the running environment (*N* = 2477)

	Model 1 (confounders, motives and attitudes, perceived environmental characteristics)		Model 2 (model 1 + perceived environmental – years of running experience interactions)	
	St. Beta (p)	SE	St. Beta (p)	SE
Constant^a^	0.258	0.237	− 0.139	0.696
*Confounders*				
Age (ref = ≥ 46 year)				
≤ 35 year	− 0.01	0.03	−0.008	0.03
36–45 year	−0.031*	0.027	−0.031*	0.027
Male (female = ref)	−0.002	0.024	−0.002	0.024
Education (higher = ref)				
Lower or middle	0.014	0.024	0.014	0.024
Years of running experience (> 5 y = ref)				
< 1 y	0.015	0.037	0.112	0.275
1–5 y	0.01	0.025	−0.023	0.166
Distance monitoring y/n (ref = no)	0.011	0.026	0.009	0.026
Watch use (ref = no)	0.025	0.037	0.029	0.037
App use (ref = no)	0.047*	0.042	0.049*	0.042
Organizational context (athletics club = ref)				
Individual	−0.016	0.032	−0.013	0.032
Friends, colleagues, small group	−0.008	0.034	−0.008	0.034
*Intrapersonal characteristics: motivations and attitudes*				
Bodily and mental advantages of running	0.041*	0.029	0.041*	0.029
Identification with running	0.017	0.025	0.015	0.025
Practical advantages of running	0.015	0.025	0.018	0.025
Individual motives for quitting	−0.022	0.017	−0.02	0.017
Social motives for quitting	0.008	0.018	0.007	0.018
*Perceived environmental characteristics*				
Hinderance by pedestrians	−0.025	0.019	−0.231	0.148
Hinderance by cyclists/mopeds	0.008	0.014	0.148	0.112
Hinderance by cars	−0.040**	0.013	0.304*	0.105
Hinderance by unleashed dogs	0.012	0.011	−0.077	0.091
Hinderance through remarks	−0.007	0.021	0.161	0.161
Hinderance through threats	−0.015	0.023	−0.167	0.179
Hinderance through poor lighting	0.016	0.009	0.023	0.073
Comfortable surface	0.037**	0.012	−0.086	0.094
Lively route	0.128***	0.013	0.127	0.097
Green route	0.686***	0.013	0.808***	0.105
*Interactions perceived environment characteristics* * years of running experience (ref = > 5 y running experience)				
Pedestrians * < 1 y running experience			0.017	0.06
Pedestrians * 1–5 y running experience			0.24**	0.041
Cyclists/mopeds * < 1 y running experience			−0.044	0.045
Cyclists/mopeds * 1–5 y running experience			−0.111	0.031
Cars * < 1 y running experience			−0.205*	0.043
Cars * 1–5 y running experience			−0.192**	0.029
Unleashed dogs * < 1 y running experience			0.046	0.038
Unleashed dogs * 1–5 y running experience			0.058	0.024
Remarks * < 1 y running experience			−0.155	0.063
Remarks * 1–5 y running experience			−0.031	0.047
Threats * < 1 y running experience			0.136	0.072
Threats * 1–5 y running experience			0.034	0.05
Poor lighting * < 1 y running experience			−0.027	0.03
Poor lighting * 1–5 y running experience			0.022	0.02
Comfortable surface * < 1 y running experience			0.099	0.039
Comfortable surface * 1–5 y running experience			0.071	0.025
Lively route * < 1 y running experience			0.076	0.039
Lively route * 1–5 y running experience			−0.1	0.027
Green route * < 1 y running experience			−0.187	0.043
Green route * 1–5 y running experience			0.04	0.029
*Model fit*				
Adjusted R^2^	0.599		0.602	
SE	0.5261		0.5244	

^a^Constant: Unstandardized Beta instead of Standardized Beta

*Significance < 0.05; **Significance < 0.01; ***Significance < 0.001

## Discussion

In this study, we investigated how perceived attractiveness and restorative capacity of the running environment can be explained by intrapersonal characteristics and perceptions of the environment and to what extent these associations differed for novice runners and more experienced runners. Our primary finding was that perceived environmental characteristics, particularly green and lively running routes and a comfortable running surface, enhanced runners’ evaluation of the attractiveness and restorative capacity of the running environment, more so than intrapersonal factors such as runners’ motives and attitudes. Perceived environmental characteristics were important to all runners and only a few differences between novice and experienced runners were found. Surprisingly, hinderance from unleashed dogs and pedestrians positively impacted the attractiveness or restorative capacity for less experienced runners.

With regard to intrapersonal characteristics, (i.e. runners’ motives and attitudes), our results showed that the level of perceived bodily and mental advantages of running and the practical advantages of running positively impacted the attractiveness and restorative capacity of the running environment. Bodily and mentally experienced advantages from the running practice, such as through the positive effects of running on health, stamina or mental relaxation, may increase the motivation and positive attitudes towards running (and the frequency of running). In addition, the practical advantages of running refer to the flexible and autonomous characteristic of running. Running can be practiced anytime, everywhere and fits easily in busy life schedules compared to other types of sports and is therefore highly valued [14, 19, 54]. This flexible and autonomous characteristic of running stimulates runners to go outside, explore new routes and environments and create favourite, attractive and relaxing running routes. However, previous positive experiences and evaluations of the attractive and relaxing environment may also stimulate motives and attitudes to go for a run. Regardless of the direction and causality of the associations found, our results show that the perceived advantages and the autonomous and flexible characteristics of running are more important determinants of perceiving the environment as attractive and restorative, than motives and attitudes such as running identity and social motivation. However, our descriptive results show that bodily and mental advantages of running and identification with running are experienced to a lesser extent in novice runners, which may impact on their evaluation of the running environment. This may also indicate that it takes some time and perseverance to fall in love with running, and thus that it is important to better understand the experiences, motives and constraints of novice runners.

Characteristics at the environmental level that were positively associated with both the attractiveness and restorative capacity of the running environment included a comfortable running surface and a lively or vivid and (mostly) green environment. These results reflect findings from previous studies showing the importance of the running surface for the enjoyment of running (e.g. soft/grass or bark running tracks are more comfortable and injury-preventive but require runners to work harder; hard/stiff and flat roads are faster but have higher risk for injuries) [19, 27, 72, 74]. The importance of running in a lively and green environment corresponds to previous findings showing positive physical and mental health benefits of these types of environments [39–44]. In addition, our results correspond with findings in the context of recreational walking, suggesting that people actively choose walking routes because of the presence of green space, which makes them more attractive and relaxed [44, 80]. Furthermore, hinderance by pedestrians and cars were negatively associated with attractiveness. Similar results were also found in the study of Ettema [36] among novice runners who took part in a ‘start to run’ programme. Hinderance by cars was also negatively associated with restorative capacity. It is conceivable that hinder by cars may go together with concerns about air pollution and breathing difficulties while running in urbanized areas, which was studied among urban recreational runners in London [81]. Because the number of years of running experience modified this association, showing that experienced runners who were hindered by cars evaluated the restorative capacity more positively than less experienced runners, it may be that more experienced runners choose to use different parts of the public space than less experienced runners. More experienced runners may prefer roads that they value because it allows them to run faster (and not because they prefer to encounter traffic). They also may run longer distances than less experienced runners, which allow them to run outside or longer outside crowded urbanized areas. The positive association we found for poor lighting on attractiveness of the running environment may also be related to the preferences for more attractive running paths and routes, for example in parks and natural areas, which are more poorly lit than public roads in urban areas.

Although runners in our sample generally evaluate their running environment very positively, our results show that some associations of environmental characteristics with perceived attractiveness and restorative capacity of the running environment differed for novice and experienced runners. For example, we found that hinderance by dogs was positively associated with perceived attractiveness of the running environment for novice runners (i.e. those involved in running for less than one year) and negatively for more experienced runners. In addition, hinderance by pedestrians was positively associated with a restorative environment among moderate experienced runners (i.e. those with one to five years of running experience). These findings indicate that less experienced runners likely perceive different environments as more attractive or restorative. They may run in parks, forests and natural areas. Such green spaces, however, attract other recreational users, such as pedestrians and dog-owners, as well. Although unleashed dogs [36] and pedestrians [27] may be a well-known constraint of runners, these constraints likely do not affect their perceived attractiveness and restorative capacity of the environment to a great extent. In addition, both a comfortable running surface and hinderance from pedestrians were positively associated with attractive and restorative capacity, respectively, among moderately experienced runners. Additionally, less experienced runners who were constrained by cars evaluated their running environment as less restorative than more experienced runners. These findings indicate that more experienced runners may choose different running environments or perceive environments differently than less experienced runners. More experienced runners may have fixed routines regarding their running routes and running locations, which are based on unconscious choices [82]. They may have chosen their running routes based on the running surface (e.g. asphalt, paved paths, pavements, unpaved paths in parks or forests, tartan or a combination between them). In addition, more experienced and serious athletes are likely more focused on their training results regarding running distance, pace and achievements and may be more motivated to run and/or are less distracted by cars and less attractive routes. They may also vary their running environments to keep the running experience more attractive for themselves. Novice runners, − who to a lesser extent showed to experience bodily and mental advantages of running and identification with running -, may more urgently need an attractive running route with lively and natural elements to encourage them more than experienced runners to regularly go for a run.

### Strengths and limitations of this study and future directions

A strength of this study is that we collected data on different levels as described by socio-ecological models [46], which allowed us to investigate intrapersonal and perceived environmental characteristics of different types of runners. In addition, our data on motives and attitudes and perceptions of the environment are based on existing literature.

This study also has some limitations. First, the Eindhoven Running Survey 2015 (ERS15) lacked geographical data on running locations, which would have allowed us to link objective Geographical Information Software (GIS)-data (on for instance running environments) to the survey data. It would be interesting to also link objective environmental characteristics of the running environment to the perceived attractiveness and restorative capacity. A potential bias that may have occurred, and we could not control for because of the missing of geographical data, is an overrepresentation of respondents living in areas with similar urbanity levels (e.g. highly urbanized or rural). Such an overrepresentation could potentially have influenced the results regarding perceived attractiveness and restorative capacity of specific running environments. Furthermore, the group of novice runners (in terms of number of years of running experience) in our sample was able to complete at least one half marathon within one year of training, which indicates a moderate level of fitness. However, we believe this has not led to a bias of the results toward more experienced runners.

Future research could focus on interrelationships between perceived environmental characteristics and objective environmental characteristics. For example, GPS-based location data on running routes, running locations and running intensity and physical activity in general could be used. In addition, from a health perspective, it is interesting to apply a longitudinal research design and follow less experienced runners for a longer time period of for instance several years to investigate running adherence and quitting patterns. To what extent do characteristics of the running environment and perceptions thereof play a role herein? How do motives and attitudes change when runners become more experienced and how is this related to their experience of the running environment?

## Conclusions

Running has become one of the most popular and practised sports and it is a well-known phenomenon in the urban streetscape, public parks and natural areas. Both scholars and policy makers increasingly have become aware that an attractively designed public space may stimulate sports participation including running. We found that perceived environmental characteristics, particularly green and lively running routes and a comfortable running surface, enhanced runners’ evaluation of the attractiveness and restorative capacity of the running environment. Perceived environmental characteristics were important to all runners, and more so than intrapersonal factors such as runners’ motives and attitudes. However, green and lively running routes, a comfortable running surface and hinderance by cars were more important to less experienced runners.

Our findings indicate that the built environment is particularly important for encouraging less experienced runners. To stimulate novice runners to stay involved in running, policy makers should prioritize the attention for the public space as the environment with the greatest potential for stimulating healthy lifestyles. It is recommended to design attractive, green and lively spaces with, for instance, separate lanes for runners and other road users. For example, governments could facilitate ‘green’ running routes by tracks - preferably with a comfortable surface - that connect parks and natural areas, while providing good access upon this green infrastructure on the neighbourhood level. Both novice runners and runners that are more experienced could benefit from this specific and low-key green running infrastructure. However, for the perseverance of running aspects including motivation and sociality and feeling of community may be important as well.

## Abbreviation

ERS15: Eindhoven Running Survey 2015
